# Dimethyl 2-methyl-1,2-dihydro­quinoline-2,4-dicarboxyl­ate

**DOI:** 10.1107/S1600536811005605

**Published:** 2011-02-19

**Authors:** Zeynep Gültekin, Wolfgang Frey, Barış Tercan, Tuncer Hökelek

**Affiliations:** a Department of Chemistry, Çankırı Karatekin University, TR-18100 Çankırı, Turkey; b Universitat Stuttgart, Pfaffenwaldring 55, D-70569 Stuttgart, Germany; c Department of Physics, Karabük University, 78050 Karabük, Turkey; d Department of Physics, Hacettepe University, 06800 Beytepe, Ankara, Turkey

## Abstract

In the crystal of the title compound, C_14_H_15_NO_4_, pairs of inter­molecular N—H⋯O hydrogen bonds link the mol­ecules into centrosymmetric *R*_2_^2^(10) dimers. These dimers are further connected *via* inter­molecular C—H⋯O hydrogen bonds, forming a three-dimensional network. The heterocyclic ring adopts a twisted conformation.

## Related literature

For the preparation of 1,2-dihydro­quinoline, see: Edwards *et al.* (1998 ▶); Yan *et al.* (2004 ▶); Petasis & Butkevich (2009 ▶); Johnson *et al.* (1989 ▶); Gültekin *et al.* (2010 ▶); Waldmann *et al.* (2008 ▶). For the biological activity of dihydro­quinolines, see: Elmore *et al.* (2001 ▶); Dillard *et al.* (1973 ▶); Muren & Weissman (1971 ▶). For the preparation of quinolines, see: Dauphinee & Forrest (1978 ▶); Yan *et al.* (2004 ▶); Tom & Ruel (2001 ▶); Tokuyama *et al.* (2001 ▶); Sarma & Prajapati (2008 ▶); Martinez *et al.* (2008 ▶); Huang *et al.* (2009 ▶); Katritzky *et al.* (1996 ▶). For the biological activity of quinolines, see: Hamann *et al.* (1998 ▶); He *et al.* (2003 ▶); LaMontagne *et al.* (1989 ▶). For hydorgen-bond motifs, see: Bernstein *et al.* (1995 ▶). For ring puckering parameters, see: Cremer & Pople (1975 ▶). For the melting point, see: Rueping & Gültekin (2009 ▶).
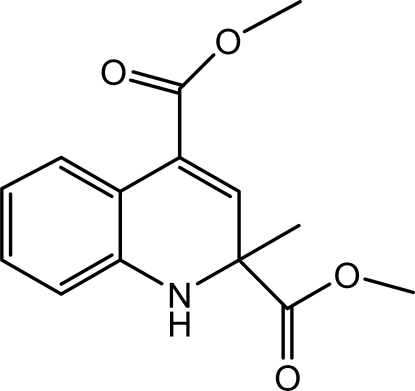


## Experimental

### 

#### Crystal data

C_14_H_15_NO_4_
*M* *_r_* = 261.27
Monoclinic, 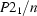

*a* = 7.9917 (12) Å
*b* = 8.8886 (11) Å
*c* = 18.9855 (18) Å
β = 99.194 (9)°
*V* = 1331.3 (3) Å^3^
*Z* = 4
Mo *K*α radiation
μ = 0.10 mm^−1^
*T* = 294 K
0.6 × 0.6 × 0.5 mm

#### Data collection

Nicolet P3 diffractometer
4144 measured reflections
3890 independent reflections
3097 reflections with *I* > 2σ(*I*)
*R*_int_ = 0.053
3 standard reflections every 50 reflections  intensity decay: 1%

#### Refinement

*R*[*F* ^2^ > 2σ(*F* ^2^)] = 0.052
*wR*(*F* ^2^) = 0.159
*S* = 1.05
3890 reflections
180 parameters
H atoms treated by a mixture of independent and constrained refinement
Δρ_max_ = 0.24 e Å^−3^
Δρ_min_ = −0.18 e Å^−3^

### 

Data collection: *XSCANS* (Siemens, 1996 ▶); cell refinement: *XSCANS*; data reduction: *SHELXTL* (Sheldrick, 2008 ▶); program(s) used to solve structure: *SHELXS97* (Sheldrick, 2008 ▶); program(s) used to refine structure: *SHELXL97* (Sheldrick, 2008 ▶); molecular graphics: *ORTEP-3 for Windows* (Farrugia, 1997 ▶) and *Mercury* (Macrae *et al.*, 2006 ▶); software used to prepare material for publication: *WinGX* (Farrugia, 1999 ▶).

## Supplementary Material

Crystal structure: contains datablocks I, global. DOI: 10.1107/S1600536811005605/bv2176sup1.cif

Structure factors: contains datablocks I. DOI: 10.1107/S1600536811005605/bv2176Isup2.hkl

Additional supplementary materials:  crystallographic information; 3D view; checkCIF report

## Figures and Tables

**Table 1:** Hydrogen-bond geometry (Å, °)

*D*—H⋯*A*	*D*—H	H⋯*A*	*D*⋯*A*	*D*—H⋯*A*
N1—H1⋯O1^i^	0.91 (2)	2.22 (2)	3.1241 (18)	174 (2)
C12—H12*A*⋯O3^ii^	0.96	2.57	3.377 (3)	142
C12—H12*C*⋯O3^iii^	0.96	2.49	3.336 (2)	148

Symmetry codes: (i) 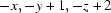; (ii) 
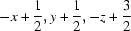
; (iii) 
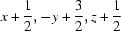
.

**Fig. 1.**
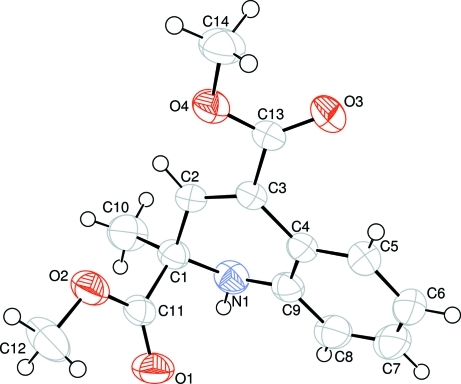
The molecular structure of the title compound with the atom-numbering scheme. Displacement ellipsoids are drawn at the 50% probability level.

**Fig. 2.**
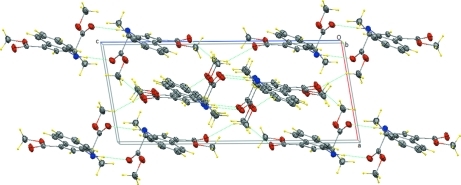
A partial packing diagram viewed down the b-axis. Hydrogen bonds are shown as dashed lines.

## supplementary crystallographic information

### Comment

Dihydroquinolines have been widely studied and found to be an important structural unit in synthetic organic and medicinal chemistry (Elmore *et al.*, 2001; Dillard *et al.*, 1973; Muren & Weissman, 1971). Many dihydroquinoline derivatives have been reported in the literature (Edwards *et al.*, 1998; Yan *et al.*, 2004; Petasis & Butkevich, 2009; Gültekin *et al.*, 2010) and some of them have biological effects. For example, 2,2,4-substituted 1,2-dihydroquinolines have been shown to possess antibacterial activities (Johnson  *et al.*, 1989). They are also important intermediates for the preparation of quinolines (Dauphinee & Forrest, 1978; Yan *et al.*, 2004; Tom & Ruel, 2001; Tokuyama *et al.*, 2001) and 1,2,3,4-tetrahydroquinolines (Katritzky *et al.*, 1996). Many synthetic methods have been developed for the preparation of quinolines (Sarma & Prajapati, 2008; Martinez *et al.*, 2008; Huang *et al.*, 2009; Waldmann *et al.*, 2008) and many quinolines display biological effects (Hamann *et al.*, 1998; He *et al.*, 2003; LaMontagne *et al.*, 1989; Muren & Weissman, 1971).

In the title compound, (I), (Fig. 1), the ring A (C1-C4/C9/N1) is not planar with the puckering parameters (Cremer & Pople, 1975) Q_T_ = 0.364 (2) Å, φ = -143.4 (3)° and θ = 113.9 (2)°.

In the crystal structure, intermolecular N-H···O hydrogen bonds (Table 1) link the molecules into centrosymmetric R_2_^2^(10) dimers (Bernstein *et al.*, 1995). These dimers are further connected *via* intermolecular C-H···O hydrogen bonds (Table 1) to form a three-dimensional network (Fig. 2).

### Experimental

The title compound was synthesized by the literature method (Waldmann *et al.*, 2008). Aniline (100 mg, 1 eq) was dissolved in chloroform (1.5 ml) in a screw-capped test tube and Bi(OTf)_3_ (5 mol%, 0.05 eq) was added to the mixture. The mixture was stirred at room temperature for 4h until the starting material was completely consumed as monitored by TLC. The resultant residue was directly purified by flash chromatography on silica (EtOAc:Cylohexane 2:98) gave in 63% yield as a yellow solid. Recrystallized over pentane and ethyl acetate (70:30) gave yellow crystalline solid R_f_ 0.53 (2:1 Cyclohexane/EtOAc) mp 346 K (Rueping & Gültekin, 2009).

### Crystal data

**Table:**

C_14_H_15_NO_4_	*F*(000) = 552
*M**_r_* = 261.27	*D*_x_ = 1.304 Mg m^−^^3^
Monoclinic, *P*2_1_/*n*	Mo *K*α radiation, λ = 0.71073 Å
Hall symbol: -P 2yn	Cell parameters from 49 reflections
*a* = 7.9917 (12) Å	θ = 17–18°
*b* = 8.8886 (11) Å	µ = 0.10 mm^−^^1^
*c* = 18.9855 (18) Å	*T* = 294 K
β = 99.194 (9)°	Block, colourless
*V* = 1331.3 (3) Å^3^	0.6 × 0.6 × 0.5 mm
*Z* = 4	

### Data collection

**Table:**

Nicolet P3 diffractometer	*R*_int_ = 0.053
Radiation source: fine-focus sealed tube	θ_max_ = 30.0°, θ_min_ = 2.2°
graphite	*h* = 0→11
Wyckoff–Scan scans	*k* = 0→12
4144 measured reflections	*l* = −26→26
3890 independent reflections	3 standard reflections every 50 reflections
3097 reflections with *I* > 2σ(*I*)	intensity decay: 1%

### Refinement

**Table:**

Refinement on *F*^2^	Secondary atom site location: difference Fourier map
Least-squares matrix: full	Hydrogen site location: inferred from neighbouring sites
*R*[*F*^2^ > 2σ(*F*^2^)] = 0.052	H atoms treated by a mixture of independent and constrained refinement
*wR*(*F*^2^) = 0.159	*w* = 1/[σ^2^(*F*_o_^2^) + (0.0757*P*)^2^ + 0.2951*P*] where *P* = (*F*_o_^2^ + 2*F*_c_^2^)/3
*S* = 1.05	(Δ/σ)_max_ < 0.001
3890 reflections	Δρ_max_ = 0.24 e Å^−^^3^
180 parameters	Δρ_min_ = −0.18 e Å^−^^3^
0 restraints	Extinction correction: *SHELXL97* (Sheldrick, 2008), Fc^*^=kFc[1+0.001xFc^2^λ^3^/sin(2θ)]^-1/4^
Primary atom site location: structure-invariant direct methods	Extinction coefficient: 0.076 (5)

### Special details

**Table:**

Geometry. All esds (except the esd in the dihedral angle between two l.s. planes) are estimated using the full covariance matrix. The cell esds are taken into account individually in the estimation of esds in distances, angles and torsion angles; correlations between esds in cell parameters are only used when they are defined by crystal symmetry. An approximate (isotropic) treatment of cell esds is used for estimating esds involving l.s. planes.
Refinement. Refinement of F^2^ against ALL reflections. The weighted R-factor wR and goodness of fit S are based on F^2^, conventional R-factors R are based on F, with F set to zero for negative F^2^. The threshold expression of F^2^ > 2sigma(F^2^) is used only for calculating R-factors(gt) etc. and is not relevant to the choice of reflections for refinement. R-factors based on F^2^ are statistically about twice as large as those based on F, and R- factors based on ALL data will be even larger.

### Fractional atomic coordinates and isotropic or equivalent isotropic displacement parameters (Å^2^)

**Table:**

	*x*	*y*	*z*	*U*_iso_*/*U*_eq_	
O1	0.16885 (15)	0.61815 (14)	0.96958 (6)	0.0632 (3)	
O2	0.15641 (17)	0.84306 (14)	0.91740 (6)	0.0671 (4)	
O3	−0.04420 (19)	0.60887 (14)	0.63398 (6)	0.0688 (4)	
O4	0.00900 (15)	0.83447 (13)	0.68455 (5)	0.0538 (3)	
N1	−0.12790 (17)	0.52602 (16)	0.88407 (7)	0.0527 (3)	
H1	−0.137 (3)	0.491 (3)	0.9282 (12)	0.080 (6)*	
C1	−0.08128 (18)	0.68333 (17)	0.88397 (7)	0.0447 (3)	
C2	−0.07661 (18)	0.73116 (16)	0.80816 (7)	0.0441 (3)	
H2	−0.1036	0.8299	0.7946	0.053*	
C3	−0.03445 (17)	0.63452 (15)	0.76014 (7)	0.0411 (3)	
C4	−0.00240 (17)	0.47523 (15)	0.77879 (7)	0.0439 (3)	
C5	0.0730 (2)	0.37209 (18)	0.73815 (9)	0.0551 (4)	
H5	0.1104	0.4042	0.6967	0.066*	
C6	0.0929 (2)	0.22295 (19)	0.75842 (11)	0.0675 (5)	
H6	0.1440	0.1557	0.7309	0.081*	
C7	0.0370 (3)	0.17430 (19)	0.81939 (12)	0.0723 (5)	
H7	0.0479	0.0734	0.8323	0.087*	
C8	−0.0351 (2)	0.27357 (19)	0.86154 (10)	0.0630 (4)	
H8	−0.0718	0.2393	0.9028	0.076*	
C9	−0.05340 (17)	0.42528 (16)	0.84272 (8)	0.0468 (3)	
C10	−0.2084 (2)	0.7764 (2)	0.91791 (10)	0.0689 (5)	
H10A	−0.2112	0.7408	0.9654	0.103*	
H10B	−0.3191	0.7667	0.8899	0.103*	
H10C	−0.1749	0.8802	0.9197	0.103*	
C11	0.09548 (18)	0.70811 (16)	0.92869 (6)	0.0432 (3)	
C12	0.3182 (3)	0.8827 (3)	0.95860 (10)	0.0782 (6)	
H12A	0.3519	0.9801	0.9441	0.117*	
H12B	0.4015	0.8094	0.9507	0.117*	
H12C	0.3087	0.8848	1.0084	0.117*	
C13	−0.02495 (18)	0.68793 (17)	0.68624 (7)	0.0455 (3)	
C14	0.0372 (3)	0.8942 (2)	0.61686 (9)	0.0639 (4)	
H14A	0.0545	1.0009	0.6209	0.096*	
H14B	−0.0598	0.8739	0.5813	0.096*	
H14C	0.1355	0.8477	0.6032	0.096*	

### Atomic displacement parameters (Å^2^)

**Table:**

	*U*^11^	*U*^22^	*U*^33^	*U*^12^	*U*^13^	*U*^23^
O1	0.0643 (7)	0.0639 (7)	0.0556 (6)	−0.0034 (5)	−0.0082 (5)	0.0187 (5)
O2	0.0860 (9)	0.0569 (7)	0.0495 (6)	−0.0208 (6)	−0.0163 (6)	0.0115 (5)
O3	0.1070 (10)	0.0603 (7)	0.0347 (5)	0.0046 (7)	−0.0018 (6)	−0.0064 (5)
O4	0.0706 (7)	0.0516 (6)	0.0369 (5)	−0.0038 (5)	0.0014 (4)	0.0041 (4)
N1	0.0563 (7)	0.0561 (7)	0.0457 (6)	−0.0088 (6)	0.0077 (5)	0.0054 (5)
C1	0.0484 (7)	0.0491 (7)	0.0360 (6)	0.0029 (6)	0.0048 (5)	0.0012 (5)
C2	0.0518 (7)	0.0421 (6)	0.0356 (6)	0.0049 (5)	−0.0014 (5)	0.0021 (5)
C3	0.0438 (6)	0.0423 (6)	0.0340 (5)	0.0002 (5)	−0.0033 (5)	0.0004 (5)
C4	0.0449 (6)	0.0411 (6)	0.0415 (6)	−0.0011 (5)	−0.0056 (5)	−0.0011 (5)
C5	0.0600 (9)	0.0502 (8)	0.0507 (8)	0.0060 (7)	−0.0046 (6)	−0.0072 (6)
C6	0.0735 (11)	0.0476 (8)	0.0732 (11)	0.0094 (8)	−0.0133 (9)	−0.0119 (8)
C7	0.0816 (12)	0.0385 (8)	0.0861 (13)	−0.0058 (8)	−0.0198 (10)	0.0041 (8)
C8	0.0688 (10)	0.0475 (8)	0.0666 (10)	−0.0150 (7)	−0.0081 (8)	0.0117 (7)
C9	0.0450 (7)	0.0444 (7)	0.0470 (7)	−0.0092 (5)	−0.0054 (5)	0.0037 (5)
C10	0.0674 (10)	0.0854 (13)	0.0559 (9)	0.0197 (9)	0.0157 (8)	−0.0024 (9)
C11	0.0522 (7)	0.0478 (7)	0.0296 (5)	−0.0014 (5)	0.0063 (5)	0.0015 (5)
C12	0.0917 (13)	0.0891 (14)	0.0472 (9)	−0.0413 (12)	−0.0086 (8)	0.0045 (9)
C13	0.0498 (7)	0.0487 (7)	0.0343 (6)	0.0046 (6)	−0.0042 (5)	−0.0008 (5)
C14	0.0774 (11)	0.0698 (11)	0.0430 (8)	−0.0016 (8)	0.0053 (7)	0.0141 (7)

### Geometric parameters (Å, °)

**Table:**

O1—C11	1.2001 (17)	C5—C6	1.382 (2)
O2—C11	1.3249 (18)	C5—H5	0.9300
O2—C12	1.443 (2)	C6—C7	1.376 (3)
O3—C13	1.2055 (17)	C6—H6	0.9300
O4—C13	1.3319 (19)	C7—C8	1.377 (3)
O4—C14	1.4410 (18)	C7—H7	0.9300
N1—C1	1.447 (2)	C8—C9	1.397 (2)
N1—C9	1.386 (2)	C8—H8	0.9300
N1—H1	0.91 (2)	C10—H10A	0.9600
C1—C2	1.5070 (18)	C10—H10B	0.9600
C1—C10	1.530 (2)	C10—H10C	0.9600
C1—C11	1.5432 (19)	C12—H12A	0.9600
C2—C3	1.3344 (19)	C12—H12B	0.9600
C2—H2	0.9300	C12—H12C	0.9600
C3—C4	1.4720 (19)	C14—H14A	0.9600
C3—C13	1.4944 (18)	C14—H14B	0.9600
C4—C5	1.395 (2)	C14—H14C	0.9600
C4—C9	1.412 (2)		
			
C11—O2—C12	116.97 (13)	C7—C8—H8	119.8
C13—O4—C14	116.33 (13)	C9—C8—H8	119.8
C1—N1—H1	112.9 (15)	N1—C9—C4	119.52 (13)
C9—N1—C1	119.30 (12)	N1—C9—C8	121.05 (15)
C9—N1—H1	114.2 (14)	C8—C9—C4	119.37 (15)
N1—C1—C2	108.63 (12)	C1—C10—H10A	109.5
N1—C1—C10	109.48 (14)	C1—C10—H10B	109.5
N1—C1—C11	110.51 (12)	C1—C10—H10C	109.5
C2—C1—C10	111.66 (13)	H10A—C10—H10B	109.5
C2—C1—C11	108.97 (11)	H10A—C10—H10C	109.5
C10—C1—C11	107.59 (13)	H10B—C10—H10C	109.5
C1—C2—H2	119.4	O1—C11—O2	123.62 (14)
C3—C2—C1	121.23 (12)	O1—C11—C1	124.78 (13)
C3—C2—H2	119.4	O2—C11—C1	111.59 (12)
C2—C3—C4	120.53 (12)	O2—C12—H12A	109.5
C2—C3—C13	119.55 (12)	O2—C12—H12B	109.5
C4—C3—C13	119.90 (12)	O2—C12—H12C	109.5
C5—C4—C3	124.94 (13)	H12A—C12—H12B	109.5
C5—C4—C9	118.56 (14)	H12A—C12—H12C	109.5
C9—C4—C3	116.50 (13)	H12B—C12—H12C	109.5
C4—C5—H5	119.4	O3—C13—O4	123.36 (14)
C6—C5—C4	121.12 (17)	O3—C13—C3	124.65 (14)
C6—C5—H5	119.4	O4—C13—C3	111.99 (11)
C5—C6—H6	120.1	O4—C14—H14A	109.5
C7—C6—C5	119.82 (18)	O4—C14—H14B	109.5
C7—C6—H6	120.1	O4—C14—H14C	109.5
C6—C7—C8	120.64 (16)	H14A—C14—H14B	109.5
C6—C7—H7	119.7	H14A—C14—H14C	109.5
C8—C7—H7	119.7	H14B—C14—H14C	109.5
C7—C8—C9	120.43 (18)		
			
C12—O2—C11—O1	−1.7 (2)	C2—C3—C4—C5	−167.06 (14)
C12—O2—C11—C1	177.32 (15)	C2—C3—C4—C9	13.20 (19)
C14—O4—C13—O3	−5.3 (2)	C13—C3—C4—C5	14.8 (2)
C14—O4—C13—C3	174.17 (13)	C13—C3—C4—C9	−164.94 (12)
C9—N1—C1—C2	44.10 (17)	C2—C3—C13—O3	−154.18 (16)
C9—N1—C1—C10	166.27 (14)	C2—C3—C13—O4	26.33 (18)
C9—N1—C1—C11	−75.40 (16)	C4—C3—C13—O3	24.0 (2)
C1—N1—C9—C4	−30.23 (19)	C4—C3—C13—O4	−155.51 (12)
C1—N1—C9—C8	152.77 (14)	C3—C4—C5—C6	−177.69 (14)
N1—C1—C2—C3	−31.14 (18)	C9—C4—C5—C6	2.0 (2)
C10—C1—C2—C3	−151.98 (15)	C3—C4—C9—N1	−0.52 (18)
C11—C1—C2—C3	89.32 (16)	C3—C4—C9—C8	176.53 (13)
N1—C1—C11—O1	−14.36 (19)	C5—C4—C9—N1	179.73 (13)
N1—C1—C11—O2	166.68 (12)	C5—C4—C9—C8	−3.2 (2)
C2—C1—C11—O1	−133.65 (15)	C4—C5—C6—C7	0.4 (3)
C2—C1—C11—O2	47.38 (16)	C5—C6—C7—C8	−1.6 (3)
C10—C1—C11—O1	105.13 (18)	C6—C7—C8—C9	0.4 (3)
C10—C1—C11—O2	−73.84 (16)	C7—C8—C9—N1	179.06 (15)
C1—C2—C3—C4	4.2 (2)	C7—C8—C9—C4	2.1 (2)
C1—C2—C3—C13	−177.68 (12)		

### Hydrogen-bond geometry (Å, °)

**Table:**

*D*—H···*A*	*D*—H	H···*A*	*D*···*A*	*D*—H···*A*
N1—H1···O1^i^	0.91 (2)	2.22 (2)	3.1241 (18)	174 (2)
C12—H12A···O3^ii^	0.96	2.57	3.377 (3)	142
C12—H12C···O3^iii^	0.96	2.49	3.336 (2)	148

Symmetry codes: (i) −*x*, −*y*+1, −*z*+2; (ii) −*x*+1/2, *y*+1/2, −*z*+3/2; (iii) *x*+1/2, −*y*+3/2, *z*+1/2.
